# Walk the line—dispersal movements of gray mouse lemurs (*Microcebus murinus*)

**DOI:** 10.1007/s00265-012-1371-y

**Published:** 2012-06-26

**Authors:** Susanne Schliehe-Diecks, Manfred Eberle, Peter M. Kappeler

**Affiliations:** 1 Courant Research Centre “Evolution of Social Behaviour”, Georg-August-University of Göttingen, Kellnerweg 6, 37077 Göttingen, Germany; 2 Behavioral Ecology and Sociobiology Unit, German Primate Center, Kellnerweg 4, 37077 Göttingen, Germany; 3 Department of Sociobiology/Anthropology, Georg-August-University of Göttingen, Kellnerweg 6, 37077 Göttingen, Germany

**Keywords:** Natal dispersal, Dispersal movements, Transfer, Translocation, *Microcebus murinus*

## Abstract

Despite the importance of dispersal for individuals and populations, little is known about the actual dispersal process in most species. We observed 90 subadult gray mouse lemurs—small, arboreal, nocturnal primates—in Kirindy Forest in western Madagascar, to determine the behavioral processes underlying natal dispersal. Twelve radio-collared males dispersed over distances between 180 and 960 m (≈1–7 home range diameters) away from their presumed natal ranges. Dispersal forays were fast and highly directed, and thus distinct from routine movements. Contrary to expectations of current hypotheses on potential differences between different types of dispersal movements, their special movement style did not prevent dispersers from interrupting forays to exploit resources they encountered during their forays. Data from a translocation experiment indicated that highly directed dispersal or search forays reflect a general strategy for large-scale exploration away from familiar sites in this species. A prolonged transfer phase was also observed, with regular commuting between old and new sites for up to 14 days, which probably served to moderate costs of unfamiliarity with a new site. In conclusion, the dispersal process of gray mouse lemurs is characterized by high intra- and interindividual consistency in movement strategies, but variation in the duration of the transfer phase. The observed dispersal movement style represents an effective strategy balancing costs of dispersal with the need to gather an appropriate level of information about potential dispersal target sites.

**Electronic supplementary material:**

The online version of this article (doi:10.1007/s00265-012-1371-y) contains supplementary material, which is available to authorized users.

## Introduction

Dispersal is a key process in individual life histories and a central topic in ecology, evolution, and conservation because it affects the fitness of individuals, determines their distribution, and has important consequences for the demographic and genetic structure of populations (Clobert et al. 2004; Nunes 2007). Dispersal is defined as a movement (active or passive) of an organism or a propagule from its site or group of origin to its first or subsequent breeding site or group (Shields 1987; Lidicker and Stenseth 1992; Clobert et al. 2009). Many animals show a sex bias in dispersal rates, with males being the predominantly dispersing sex in mammals (Greenwood 1980). In the past years, much effort has been put into studying the influence of inbreeding avoidance and competition for resources and mates on the evolution of sex-biased dispersal, the associated costs and benefits of dispersal for individuals (but also populations), and how they vary in space and time (Greenwood 1980; Dobson 1982; Waser et al. 1986; Pusey and Packer 1987; Smith 1987; Alberts and Altmann 1995; Isbell and Van Vuren 1996; Dufty and Belthoff 2001; Andreassen et al. 2002; Pasinelli et al. 2004; Yoder et al. 2004; Boinski et al. 2005; Bowler and Benton 2005; Nunes 2007; Ronce 2007; Bonte et al. 2012; Clutton-Brock and Lukas 2012).

However, our understanding of dispersal is limited because for many species we lack detailed information on the behavioral processes underlying different phases of dispersal (emigration, transfer, and immigration). Because of the practical difficulties of observing this often once-in-a-lifetime event, studying the transfer phase of dispersal remains especially difficult. Systematic analyses of the possible advantages and disadvantages of different transfer strategies are therefore mainly of a theoretical nature because they rely on modeling of the transfer phase of dispersal under different assumptions and conditions, mainly in a patchy landscape matrix. For instance, some of these studies investigated the effectiveness of different movement strategies (random and systematic searches), or they explored which type of movement develops under a given set of conditions (Zollner and Lima 1999; Wiens 2001; Conradt et al. 2003; Heinz and Strand 2006; Barton et al. 2009). However, there is a gap between modeling approaches and empirical studies in that the number and sophistication of theoretical models of dispersal far exceeds our knowledge of actual animal movements. First approaches to generate working hypotheses to evaluate empirical dispersal movements differentiate between dispersal via routine movements or special movements (Van Dyck and Baguette 2005). If dispersal is accomplished by special movements, it is proposed that the movements should differ from routine movements, in general features like spatial scale of displacement, speed of movements, configuration of trajectory, and responses to conspecifics and resources during movement.

In the present study, we investigated dispersal behavior and movements of the nocturnal, solitary gray mouse lemur (*Microcebus murinus*, J.F. Miller 1777) in the Fôret de Kirindy, a dry deciduous forest in western Madagascar (Eberle and Kappeler 2004a, b). Gray mouse lemurs are small (60 g), omnivorous primates and can be found in most remaining forests in southern and western Madagascar (Mittermeier et al. 2010). Despite their solitary activity, individual home ranges of about 1.5 ha overlap extensively between and within sexes. This fact distinguishes gray mouse lemurs from most other species for which data on the dispersal process are available (Bearder 1987; Mech 1987; Steen 1994; Estes-Zumpf and Rachlow 2009). During the day, closely related females form stable sleeping groups, whereas adult males only occasionally share sleeping sites. For the duration of the short annual mating season, males roam widely, more than quadrupling their habitual home range. After 2 months of gestation, females give birth to one to four young, which are weaned at the age of about 2–3 months (Schmid 1998; Schmid and Kappeler 1998; Fietz 1999; Radespiel 2000; Eberle and Kappeler 2002, 2004a, b).

Previous population genetic studies revealed that dispersal in gray mouse lemurs corresponds to the general mammalian trend of male-biased natal dispersal (Eberle and Kappeler 2002; Radespiel et al. 2003; Eberle and Kappeler 2004a, b; Fredsted et al. 2004, 2005). With the onset of the austral fall, subadult males start to disperse (Eberle and Kappeler 2004a; Kraus et al. 2008). However, the actual behavioral processes generating this distribution of individuals remain unknown. Therefore, the main focus of our study was to investigate how gray mouse lemur males disperse and to describe different aspects of the dispersal process. If dispersal is achieved through a particular type of movement, potential differences to routine movements include a high degree of linearity of movement pathways, high movement speed, and no response to resources or conspecifics (Van Dyck and Baguette 2005). Below, we portray (high resolution) dispersal movements and contrast them with normal movements as well as roaming movements during the mating season, to investigate whether dispersing gray mouse lemurs adopt a behaviorally different movement style during dispersal. We also present results of a translocation experiment whose aim was to explore how gray mouse lemurs move in unfamiliar habitats and whether they were able to successfully home back to their usual home range. Finally, to further test whether gray mouse lemurs alter their movement behavior during dispersal, we compared travel distances of mouse lemur males during different situations (routine, dispersal, roaming, and translocation).

## Methods

### Study site and capture

The study was conducted within a 12,500-ha forestry concession of the Centre National de Formation, d'Etude et de Recherche en Environnement et Foresterie (CNFEREF) de Morondava in Kirindy Forest. This dry deciduous forest is situated 60 km northeast of Morondava in western Madagascar (44°39′ E, 20°03′ S). The region is characterized by pronounced seasonality with a single rainy season between December and March and a dry season from April to November (Kappeler and Fichtel 2012). The study took place in a 60-ha area, locally known as CS7, containing a rectangular system of small foot trails at 25- to 50-m intervals (described in Eberle and Kappeler 2004a). For the translocation experiment, data were additionally collected within a second grid system, locally known as CS5 (26 ha, described in Lührs et al. 2009).

### Data collection and processing of dispersal, normal, and roaming movements

Gray mouse lemurs in CS7 have been continuously captured, marked, and studied since 1994. In order to collect behavioral data for this study, we captured subadult individuals and supplied them with radio collars (Holohil Systems Ltd., BD-2C, 1.8 g). Trapping procedures and animal handling followed the protocol described in Eberle and Kappeler (2004a). Individuals were classified as subadult by their small body mass (<55 g), small size, and the absence of a subdermal passive transponder. Trappings were conducted on three consecutive nights once per month in a central capture area (9 ha, 180 traps) and twice per year in the surrounding areas (25 ha, 210 traps). Altogether, we equipped 90 subadult individuals with radio collars, 28 females and 62 males. We detected no obvious signs of adverse effects of the radio collars on individuals. At the end of the study, an attempt was made to remove all radio collars. To this end, we conducted special, targeted trapping sessions for dispersers which had left the study area. Twelve individuals (two females, ten males) were not recaptured at the end of the study. One female was only recaptured a year after this study, and she showed no signs of adverse effects caused by the radio collar.

Data of dispersal movements were collected during the dry seasons between March and June 2007, March and May 2008, April and September 2009, and April and September 2010. Between 1800 and 2400 hours, we determined locations of radio-collared animals between one and three times per night. Data points were considered to be statistically independent of each other if they were collected at least 20 min apart (Dammhahn and Kappeler 2009). If individuals were sighted, their exact position was determined with reference to the trail system or with the help of a GPS device (Garmin GPSMAP 76CSx, accuracy of position <10 m RMS). When individuals were not visible, their location was determined via triangulation to the nearest 25 m. Spatial data were recorded as UTM coordinates and processed and visualized in ArcGIS 9.3 (Esri) and the toolbar “Home Range Tools” for ArcGIS® (Rodgers et al. 2007). If an animal moved outside its regular home range, we tracked this particular individual continuously in order to determine direction and distance of movements in detail.

Number of independent data points for calculation of regular home ranges and the time period during which they were collected varied between individuals because of predation events, variation in the life span of radio collars, length of field season, and dispersal events. As a result, these spatial data were collected over periods ranging from 1 week up to 7 months. Dammhahn and Kappeler (2009) determined that 50 independent locations are sufficient to calculate representative minimum convex polygons (MCP) of gray mouse lemur's home ranges. If we had more data points per individual, we reduced them to a random sample of 50 points, to balance sample size among individuals. Control calculations of home range location using all available data for individuals with more than 50 data points revealed stability of all home range positions independent of sampling period.

### Data collection and processing of movements of translocated individuals

Translocated individuals were captured during additional, targeted capture sessions in smaller areas, using about 40 traps. These animals were trapped twice within 5 days, once to translocate them from their familiar range for the experiment and again to return them to their familiar area. We weighed them before and after the experiment to check their health status. Following the experiment, we kept mouse lemurs for one night at the research station and supplied them with bananas and raisins.

Six males were translocated for a period of 3 days between August and September 2010. Translocation distances ranged from 0.2 to 2 km, equaling 1 to 14 home range diameters. All six translocated individuals originated from the grid system CS7, and except for one individual, all were translocated within CS7. For these five individuals, we knew their natal home range, so we could ensure that we could transfer them to an unfamiliar area within the same grid system. They were translocated over 200 and 600 m (about one to three home range diameters). The remaining individual was transferred to another grid system (CS5) 2 km away because we did not know its natal origin. Because 2 km is well above the maximal observed dispersal distance, this strategy ensured that we translocated this individual to an unfamiliar area where we could also observe it more easily.

To release the animals, we positioned traps with the trap door closed on branches at about 1.5 m height at around 1745 hours. At 1800 hours, we opened the trap door. From that moment, we recorded every minute the whereabouts of the animal and their behaviors (e.g., feeding, interactions, etc.) via one–zero sampling (Martin and Bateson 1993). The animals decided on their own account when they would leave the trap (5- to 60-min latency). Every animal was released at a different position. We observed one individual at a time and followed it over the whole activity period from dusk till dawn for the three nights, yielding between 1,380 and 1,815 location points per individual (median = 1,784) recorded during focal observations. Contact time during observations varied between 68 and 84 % (median = 82 %) because the focal animals were occasionally out of sight. On the fourth evening, animals were retrapped (see above) to return them to their habitual home range.

We calculated the size of prevailing ranges (area used by translocated individuals) as 95 % kernels for each night separately, using independent locational data points (20-min intervals) from the period when an individual was active. The size of these areas was compared using Friedman's test of the “stats” package (© R Core Team and contributors worldwide). Pairwise comparisons between nights were conducted using a paired Wilcoxon rank sum test, and α levels were adjusted for multiple testing using the Bonferroni correction (Rice 1990). Results of statistical tests and a figure of prevailing range sizes were generated with the software R vers. 2.14.2 (© The R Foundation of Statistical Computing).

### Average travel distances per hour

To test whether sex and different circumstances (routine, dispersal, roaming, and translocation) affect movement behavior, we compared average travel distances. Data on routine movements within the home range were based on focal observation of 19 subadult females (no. of observations, 1–27) and 36 subadult males (no. of observations, 3–22) collected between 18 and 23 h during March and May 2008, April and September 2009, and April and September 2010. Four animals per night in changing order and combinations were observed for 40-min intervals. Data on roaming movements originated from focal observations of 19 males (no. of observations, 1–16) from October to November 1999 to 2001. One to nine males per night were observed (50–60 min) by one to three observers. Travel velocities during dispersal and translocation were calculated for six individuals. Mean standardized hourly travel distances per individual were computed. To do so, we calculated the average distance moved per minute during observation bouts and extrapolated these distances up to hourly distances. We compared travel distances during routine movements of subadult males and females using an unpaired Wilcoxon rank–sum test to determine whether males differ from females in general. To test for equality of male travel distances in different situations (routine, dispersal, roaming, translocation), we compared them separately using Kruskal–Wallis rank sum test and Nemenyi–Damico–Wolfe–Dunn test, also known as Dunn's post hoc test (Hollander and Wolfe 1999). For Dunn's post hoc test, exact *p* values and 99 % confidence interval were approximated via Monte Carlo resampling based on 90,000 permutations. Graphics were generated and statistical tests computed with the packages “coin” (Hothorn et al. 2008b) and “multcomp” (Hothorn et al. 2008a) in R vers. 2.14.2 (© The R Foundation of Statistical Computing).

## Results

### Dispersal movements

**Fig. 1:**
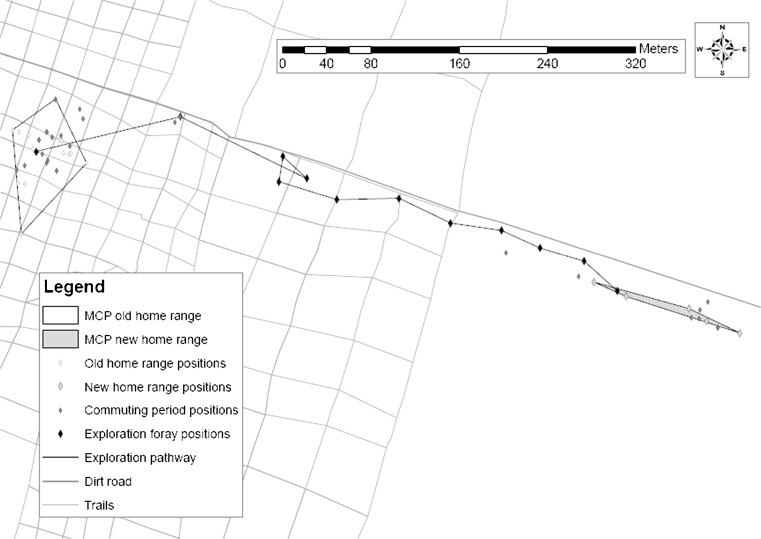
First exploration foray and commuting of successful disperser 22F7: an example of a typical dispersal event. The figure shows a section of the grid system. Different symbols indicate different phases of the dispersal process. The *black line* indicates the pathway during the first exploration foray. The 95 % MCPs for old and new home range were calculated based on statistically independent locations. We used 50 randomly chosen data points (*white diamonds*) for the old home range, but had only five locations for the new home range (*big*, *light gray diamonds*). Positions taken during the commuting period (*small*, *dark gray diamonds*) were collected on different days and thus depict no movement path. They are shown here to illustrate the fidelity of the disperser to its dispersal foray during the commuting phase. These positions were not included into MCP calculations

Dispersal movements differed from “routine” movements. Dispersal was characterized by a distinct displacement of the home range, which was accomplished through highly directed movements (Fig. 1). None of the 28 females ever made attempts to relocate their home range, but for 12 out of 62 males, we were able to collect data on details of the dispersal process (duration of the transfer phase, distance). For the remaining 50 males, which did not shift their home range, 19 died due to predation. However, parentage analyses suggested that most of these 50 males were immigrants when they were captured the first time (unpublished data). These population-wide parentage analyses were highly effective for females, assigning about 80 % of them to mothers, but failed to detect mothers for 75 % of the males.

**Fig. 2:**
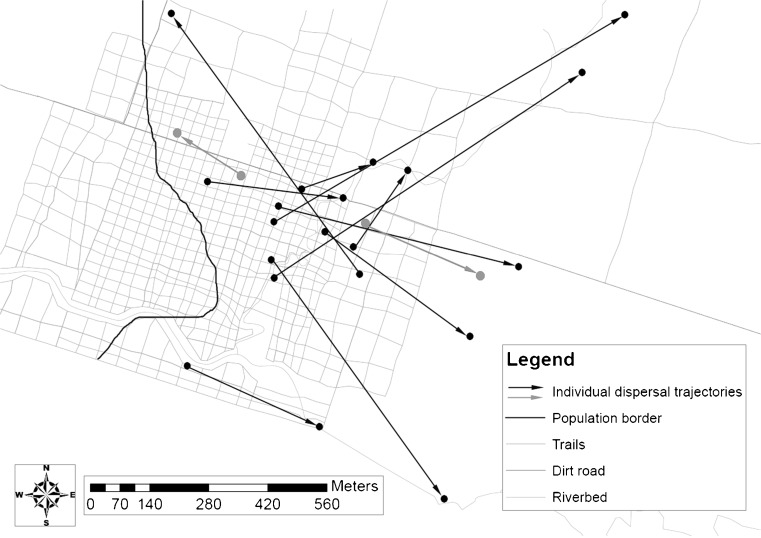
Directions and distances of individual dispersal trajectories. Illustrated are the directions and distances of dispersal for *n* = 12 male dispersal events (dispersal trajectories of successful dispersers are depicted in *black*; unsuccessful dispersers are depicted in *gray*). *Arrows* indicate the direction of dispersal and point from the central position of the old home range of a given individual towards the new home range. The *black line* sketches the natural border of our population, where, to the western side, population density strongly decreases. For the other directions, the total extent of the population remains unknown so far

Ten of the 12 males dispersed “successfully” in the sense that they relocated their home range to a new site. We never observed them prospecting another site afterwards, suggesting that secondary dispersal is rare in this species. These males dispersed over distances ranging from 180 to 960 m between April and June, except for two males, which dispersed during the mating season in October/November (220- and 350-m dispersal distance, respectively). The two unsuccessful dispersers remained in their natal area after initial dispersal trials in June and August. Their forays away from their natal home range had a distance of 100 and 280 m (Online resource 1, 2). Individuals showed no obvious behavioral signs of imminent departure and did not seem to face increased aggression prior to and during dispersal. How dispersers chose where to go and where to stay remains unclear. There were no obvious landscape features that might have guided or constrained dispersal direction in this continuous forest. However, dispersers seemed to choose to disperse more frequently towards the eastern and northeastern part of our study site (2 out of 12; Fig. 2). Unfortunately, we lack data on habitat structure and population densities for the settling areas, so that the actual mechanisms of habitat selection remain unknown. Transect captures and sightings of conspecifics during the night indicate that all dispersers transferred through and to areas inhabited by other gray mouse lemurs. Another indication that conspecific presence might be important comes from observations of one successful disperser that dispersed in the direction of a natural border of our study population in the northwestern part of our study area, where population density decreased markedly (Fig. 2). On the night of dispersal, this male moved beyond that edge, but we do not know the full extent of its foray because the transmitter signal was lost. The next morning, he had returned to the periphery of the study population, where he subsequently established his home range.

The duration of the transfer phase varied between individuals (range, 1–14 nights), but all observed dispersal distances could be covered within one night. Four successful dispersers completed dispersal within one night (distance between old and new home ranges, 320 and 830 m). They exhibited one directed movement towards their new home range and never returned to their old home range. For six individuals (four successful, two unsuccessful dispersers), the process lasted between 7 and 14 nights. These animals moved back and forth between their old and new home range, sometimes even within the same night (“dispersal forays,” see below). For the remaining two individuals which dispersed during the mating season, we lack information on the duration of the transfer phase and could only establish that they had relocated their home range after the end of the mating season.

For 6 of the 12 males (four successful, two unsuccessful dispersers), we obtained additional detailed information on dispersal movements (Fig. 1, Online resource 1, 2). Dispersal forays were highly directed movements away from the natal area and differed strongly from roaming and routine movements within the home range (Online resource 3, 4), which usually do not exhibit such a high degree of linearity. In cases where the transfer phase lasted longer than one night, dispersal forays occurred always in the direction where successful dispersers would ultimately establish their new home range (Fig. 1). Like successful dispersers, unsuccessful dispersers also never changed the destination of their dispersal forays to investigate other areas (Online resource 1, 2). Commuting between the old and new home range could occur on a daily basis. During the transfer phase, an individual might also leave for several nights and then return to its old home range for several nights, having sleeping sites within both areas (Fig. 1, Online resource 2). Movements back to the natal range were also highly directed (Online resource 2). Remarkably, we observed these individuals to move considerable distances (about 10 m) continuously on the ground at high speed, something we never observed during routine movements. However, if animals encountered food resources (insects, gum) during forays, they did not forego their exploitation (Online resource 2).

### Translocation movements

**Fig. 3:**
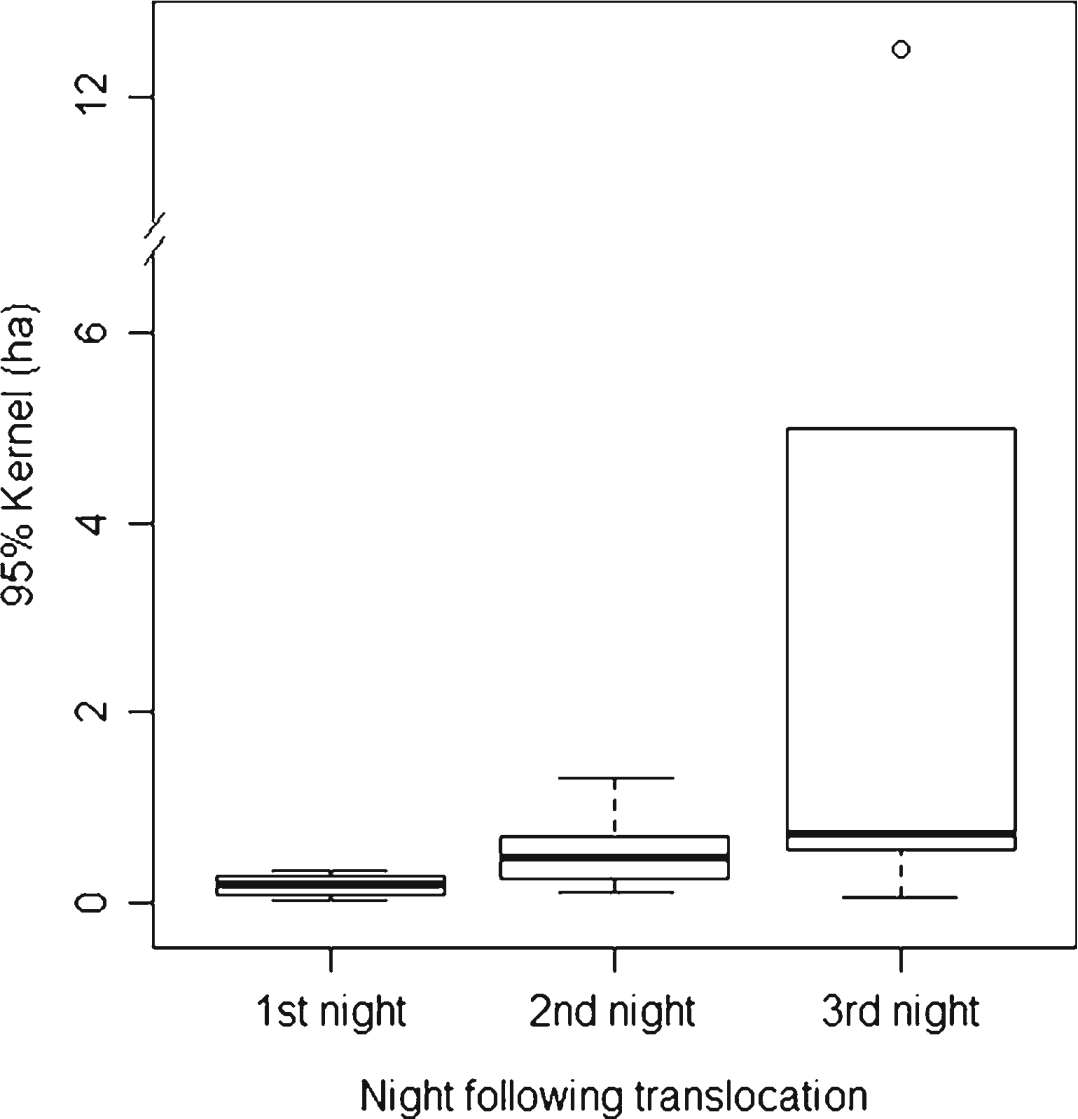
Size of prevailing ranges of six translocated males calculated as 95 % kernel (in hectares). Shown are median, interquartile range, max–min range, and outliers. Size of home ranges was calculated based on temporally independent positions, collected for the respective nights. Prevailing ranges increased over time. A habitual home range of a subadult gray mouse lemur encompasses an area of about 1 ha

Translocated individuals did not walk randomly in space, but established “prevailing ranges.” These prevailing ranges increased gradually in size during the first night of translocation and again during the following two nights (Friedman rank–sum test: *χ* ^2^ = 6.33, *df* = 2, *p* = 0.042; Fig. 3, Online resource 5, 6). Two-sided pairwise comparisons did not reach the adjusted critical α level of 0.017 (first and second nights: *V* = 0, *p* = 0.03; first and third nights: *V* = 1, *p* = 0.0625; second and third nights: *V* = 7, *p* = 0.56).

During the first night, translocated males remained close to the area where they were released (Online resource 5, 6). This pattern changed during the following two nights, and individuals traveled less often to the release position. Starting from the second night of translocation, five of six males made spacious forays, which strongly resembled the highly directed movements during dispersal forays (Fig. 1, Online resource 1, 2, 5, and 6). Maximum linear distance of these homing forays was 600 m. Only one male returned to his habitual home range during the third night of translocation (Online resource 6), but he was only translocated over a distance of about one home range diameter (200 m). When the translocated male came close to the border of the home range of the neighboring individual during his second spacious foray, he changed direction and went directly back to his home range, traversing the home range of the apparently familiar individual (Online resource 6).

### Average travel distances per hour

**Fig. 4:**
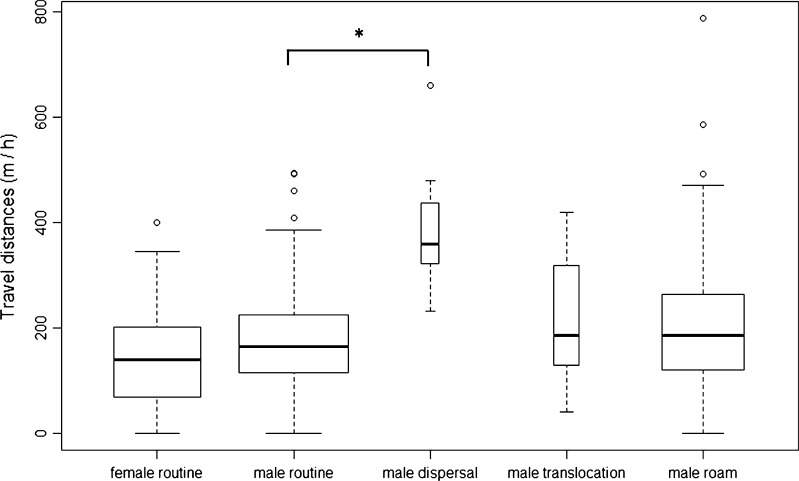
Standardized travel distances per hour for females during routine movements and males during different situations. For a comparison of travel distance, we used 204 observations for routine movements of subadult females (*n* = 19), 329 observation bouts of subadult males (*n* = 33), 9 observations of male dispersal pathways (*n* = 6), 188 observations on male roaming pathways (*n* = 19), and 18 data points of translocated males (*n* = 6). Shown are median, interquartile range, max–min range, and outliers; width of boxes indicates sample size. The *bracket* and *star* mark the significant difference between pairwise comparison of routine and dispersal movements. Remaining pairwise comparisons between male and female routine movements on the one hand and between male movements during different situations on the other hand revealed no differences.

Subadult males and females did not differ in average routine travel distances (Fig. 4, *W* = 322, *p* = 0.24). During the first half of the night in the dry season, subadult males moved on average 203 m/h ± 64 m and subadult females about 183 m/h ± 77 m. However, males did not move with the same travel velocity in all conditions (Fig. 4, Table 1, Kruskal–Wallis *χ* ^2^ = 19.64, *df* = 3, *p* < 0.001). In fact, males doubled their travel distance per hour during dispersal (mean, 405 m/h ± 136 m) in comparison to routine movements (Table 1, *p* < 0.001).

**Table 1:** Results of Dunn's post hoc comparison for average travel distances per hour for males

	Dispersal	Routine	Roaming	Translocation
Dispersal	–			
Routine	6.67E−05*	–		
Roaming	0.36	0.17	–	
Translocation	0.08	0.04	0.99	–

Global *p* value, *p* = 6.67E−05; 99 % confidence interval, lower boundary = 1.707713E−05, upper boundary = 1.739870E−04

## Discussion

Dispersal has been considered as a key behavioral mechanism in population biology and evolution, but it remains poorly documented in most species. Despite the unpredictability of this event and the practical difficulties of studying dispersal in a small, nocturnal, and arboreal mammal, we were able to collect data on behavioral aspects of natal dispersal for 12 individuals. Dispersal distances ranged from about one to seven home range diameters, and dispersal forays were behaviorally distinct from movements in other situations. Transfer was observed to last up to 14 days. Experimentally translocated individuals used the same linear movement strategy as during dispersal (homing forays). These data also imply that spatial knowledge of subadult gray mouse lemurs might be restricted to the familiar home range and that they lack a compass and or a map, because individuals that were translocated over short distances were not able to home back directly after release. Directions of dispersal and homing forays occurred in all cardinal directions. These aspects are discussed in detail below.

### What determines maximum dispersal distances?

Knowing a species' range of possible dispersal distances is important for understanding the dynamics within and between populations. Short-distance dispersal is frequent and influences the composition of many attributes of a population, like abundance or relatedness, whereas long-distance dispersal seems to occur at lower frequencies and plays an important role in interpatch dispersal, recolonization, and invasion processes (Smith 1987; Koenig et al. 1996; Sutherland and Harestad 2000). During this study, individuals were able to relocate their home range within one night or in one movement step, overcoming distances of 180–960 m. This distance range corresponds to the maximum dispersal distances detected by capture–recapture studies (Radespiel et al. 2003; Fredsted et al. 2005). Also, our results correspond to predicted dispersal distance based on a study investigating the influence of body mass and home range size on maximum possible dispersal distance (Bowman et al. 2002). However, whether the model can be used to predict maximum dispersal distance has still to be evaluated, because reported dispersal distances do not always match predicted ones. Reported dispersal distances of two other nocturnal primates, the Southern lesser bushbaby (*Galago moholi*; body mass, 100–250 g; home range size, 7–11 ha) and the slow loris (*Nycticebus coucang*; average body mass, 685 g; home range size, 0.6–15 ha), correspond to predicted values. Southern lesser bushbaby males dispersed over distances of 2,000 m in four to five consecutive nights, which is typical for many territorial species, since dispersers have to search for vacant territories and therefore move on if they cannot establish themselves in a certain area (Bearder 1987). Slow lorises dispersed up to 3,000 m (Wiens and Zitzmann 2003). However, root voles (*Microtus oeconomus*; average body mass, 50 g; reported max. dispersal distance, 3.2 km), a terrestrial mammal of similar size to gray mouse lemurs, and pygmy rabbits (*Brachylagus idahodensis*; average body mass, 500 g; reported max. dispersal distance, 6 km) exceed maximum dispersal distance predicted for animals of their respective size by far (Steen 1994; Estes-Zumpf and Rachlow 2009). Thus, to determine what finally determines maximum dispersal distance, more observational data, capture–recapture, or genetic studies with appropriate size of study area and sampling intensity are required.

### Movements in unfamiliar areas: dispersal and homing forays

Dispersal movements differed considerably from routine or roaming movements. Dispersing gray mouse lemurs followed a rather straight line and covered larger distances per hour than during routine movements, while moving through unfamiliar habitats. Direction and distance of dispersal forays were consistent within individuals but differed between dispersers. Most strikingly, translocated gray mouse lemurs episodically fell back to the same mode of movements during translocation, which we interpreted as homing forays (cf. Lührs et al. 2009). Therefore, we think that these fast, directed movements are the standard exploration or dispersal strategy in gray mouse lemurs. Contrary to expectations (Van Dyck and Baguette 2005) that specialized dispersal movements do exclude dispersers from reacting to external cues, they did exploit resources during dispersal forays, even though they used a behaviorally distinct movement strategy to disperse. In combination with the fact that the presence of conspecifics plays a prominent role in the process of habitat choice (Stamps 2001), we think this working hypothesis should be revised to incorporate the possibility that decisions of dispersers are influenced by cues they encounter during transfer, even if they use special dispersal movements (Clobert et al. 2009). Comparable rapid straight line excursions have also been described for other species. Southern lesser bushbabies moved in a highly stereotypic, directed fashion during dispersal (Bearder 1987), and translocated males of this species also fell back to the same stereotypic movement mode (S. Bearder, personal communication). Other examples come from carabid beetles (Baars 1979), butterflies (Baker 1969), wolves (Mech 1987), red foxes (Storm et al. 1976), black bears (Rogers 1987), ground squirrels (Holekamp and Sherman 1989), and slow lorises (Wiens and Zitzmann 2003). Therefore, a variety of territorial and nonterritorial taxa with different types of social organization adopt this movement strategy during dispersal. But what could be the adaptive value of highly directed rapid dispersal movements?

One possible explanation for the observed dispersal movements of gray mouse lemurs is that linear movements represent an effective search strategy that offers reasonable information gain during transfer while balancing potential costs of dispersal movements. This assumption is based on theoretical models in which almost linear movements were the most adaptive and effective random searches, especially in situations with a high degree of landscape uniformity, high predation risk, and limited energy reserves (Zollner and Lima 1999; Conradt et al. 2003; Heinz and Strand 2006). Gray mouse lemurs conformed to some of these conditions, because predation risk is high for gray mouse lemurs (Rasoloarison et al. 1995) and they inhabit a range of different habitats (Rasoloarison et al. 2000). Moreover, concerning the gain of information, translocated individuals used the same movement strategy during homing forays to cover larger distances within unfamiliar areas. Therefore, observed dispersal movements might have evolved to balance dispersal costs and the need to gather information. However, the results of these models have to be transferred to gray mouse lemurs with caution because some of the underlying assumptions do not closely reflect the situation of a dispersing mouse lemur. These models often have to rely on unrealistic assumptions about costs and risks of dispersal, like an increased risk of starvation or predation, because they measure effectiveness of dispersal strategies as success probability of finding a suitable patch (Conradt et al. 2003). For gray mouse lemurs, the transfer phase can be an event of a few hours, so starvation is an unlikely cost. Instead, the benefits of getting familiar with the new site might be much more important for the fitness of gray mouse lemurs because of seasonality of food availability (Dammhahn and Kappeler 2008, 2009; Lührs et al. 2009).

Alternatively, the question arises whether gray mouse lemurs need an elaborate systematic search strategy. A more parsimonious explanation might be that moving straight away from the natal range is a simple strategy to distance oneself from relatives in order to avoid inbreeding. Linear movements could allow individuals to cover larger distances in comparison to systematic search (Conradt et al. 2003), and they are feasible in a seemingly continuously suitable landscape. However, separation from relatives does not explain the variation in observed dispersal distance, and short-distance dispersers still lived close to their female relatives. Also, if linear movements were to present a lowly elaborated movement strategy which was not subjected to evolutionary trade-offs, it still remains difficult to explain how dispersers deal with the fact that the distribution of gray mouse lemurs is not homogeneous in continuous available habitat (Fredsted et al. 2004). It therefore seems unlikely that there are no further benefits connected to this movement strategy.

Our data on translocation movements point towards another possible constraint that might favor the observed dispersal strategy. For gray mouse lemurs, spatial knowledge of subadults might be restricted to their own home range because even over very short translocation distances, they were unable to home directly back to their habitual home range. Prevailing areas during translocation increased for all individuals from the first to the second and/or third night with decreasing frequency of moving to the area of release, indicating that spatial knowledge is accumulated by moving through a certain area (Fig. 3). With increasing experience with a site, the navigational abilities and effectiveness of an individual probably also improve (Joly and Zimmermann 2011). A study on the spatial memory abilities of gray mouse lemurs (Lührs et al. 2009) suggested that they have a mental representation that is more detailed than a network of routes and landmarks, referred to as route-based network (Byrne 1979). This way of orientation relies strongly on experience, because individuals need to form a spatial representation of traversable paths. For dispersal movements, this form of orientation could restrict the degree of tortuosity of dispersal movements if tortuosity compromises the option of returning to the old home range. Because gray mouse lemurs mark their home range with saliva, anal secretions, and urine, dispersers may use these odors as a source of information guiding their movements (Schilling 1979). Conspecifics' or individuals' own scent marks could also serve as landmarks that facilitate route reversal.

### Why commute?

Commuting between old and new home range during the transfer phase seemed to play an important role for some gray mouse lemurs during dispersal. Some individuals extended their transfer phase up to 14 days. Extended periods of commuting are also known for other species, like European badgers (Roper et al. 2003) or dwarf mongooses (Rood 1987). We think the advantage of commuting between two areas is the possibility to explore the physical and social settings of the new site while still relying on resources at the old site. The individuals of our study population mainly feed on gum during the time of dispersal (Dammhahn and Kappeler 2008). Since they have to learn where to find gum trees in the new home range, it seems advantageous not to abandon the old home range before having gained enough knowledge about the new site, especially when competing with residents for these crucial resources (Génin 2003; Lawson Handley and Perrin 2007; Del Mar Delgado et al. 2009; Lührs et al. 2009). Also, individuals responded to resources that they encountered on their forays (Online resource 2). Therefore, commuting is one option for dispersers to reduce costs or avert fatal consequences of dispersal.

### Dispersal direction

Directions of dispersal and homing forays in gray mouse lemurs occurred in all cardinal directions. However, there seemed to be a higher preference to disperse towards the eastern and northeastern part of our study site. This tendency needs to be corroborated through additional data, analyzing the relationship between gradients of habitats and population densities for gray mouse lemurs and dispersal directions. Constraints on dispersal directions on a population level have also been described for pygmy rabbits, where landscape features limit directions (Estes-Zumpf and Rachlow 2009).

Concerning direction of individual dispersal trajectories, at this point, it remains unknown how gray mouse lemur males decide in which direction to go and where to stop during dispersal or homing forays. Though unlikely, we cannot fully exclude the possibility that subadult males made undetected dispersal forays or explored the surroundings of their natal home range, as has been described for North American red squirrels (*Tarniasciurus hudsonicils*) (Larsen and Boutin 1994). Direction of dispersal can also be chosen spontaneously without prior exploration of alternative options (Smith 1974). If dispersers chose direction randomly, it remains unclear why individuals become inflexible once they have chosen a direction, because unsuccessful dispersers never changed the direction of their dispersal forays. One option for individuals to modulate dispersal success could then be to adjust dispersal distances whenever they encounter a gradient in habitat suitability. Our observation of a disperser that moved first beyond the point where it established its home range and subsequently reduced linear distance between old and new home range might indicate such a modulation.

## Conclusions

Although there has been much progress in identifying general dispersal trends via genetic studies, species or individual dispersal abilities and processes have virtually remained a black box. We need further empirical studies of a variety of species that differ with respect to their social organization and spatial tolerance to better understand how dispersal strategies and distances evolved. The present study is one of only a few describing detailed dispersal movements under natural conditions. The prominence of highly directed movements in a variety of taxa suggests general advantages of this dispersal strategy. One advantage of this movement strategy seems to be the opportunity to commute between old and new home ranges relatively easily, which in turn allows dispersers to mitigate costs of unfamiliarity. Thus, unsuccessful dispersal does not necessarily have fatal consequences, which is usually assumed in modeling approaches.

## Electronic supplementary material

Below is the link to the electronic supplementary material.

ESM 1 Exploration forays of unsuccessful disperser C404. **a** First recorded exploration foray. The figure shows a section of the grid system. The natal home range of the individual is indicated by a 95 % MCP, which was calculated based on 50 temporally independent locations (*white diamonds*) chosen randomly from 196 data points. *Gray diamonds* show recorded positions during the exploration foray, which we attempted to collect every minute (no. of positions, 127; observation time, 148 min). The *solid line* connecting them shows the pathway. **b** Second recorded exploration foray. The second exploration foray occurred 1 week after the first exploration foray. *Gray diamonds* show again positions during the exploration foray, and the *solid line* shows the pathway of the second exploration foray (no. of positions, 76; collected within 84 min). The *dashed, gray line* indicates the route for the first exploration foray. (JPEG 346 kb)

ESM 2 Return pathways of unsuccessful disperser 4B8E. **a** First recorded return pathway. The figure shows a section of the grid system. The natal home range of the individual is indicated by a 95 % MCP, which was calculated based on 50 temporally independent locations (*white diamonds*) chosen randomly from 75 data points. The *solid line* connecting the *gray diamonds* shows the return pathway (no. of positions, 185). The individual had slept at the prospected site (*black star*) and was followed from the moment when it started its activity. After initial exploration of the new area, the animal eventually returned to its natal area. *Gray crosses* represent positions where the individual stopped to feed on gum. The individual was found to have returned back to the prospected site within the same night. **b** Second recorded return pathway. The second return pathway was recorded the following night. After returning to the prospected area the night before, it used the same sleeping site (*black star*). Again, we followed the individual from the moment activity started (no. of positions, 81). This was the last incident during which this individual was observed to move outside its natal area. (JPEG 290 kb)

ESM 3 Roaming pathway of a 1-year-old male during mating season. The figure shows a section of the grid system. Illustrated is a pathway of a 1-year-old male during mating season in the year 2000. The male appeared at our study site in April 2000 and was present until October 2003. In contrast to dispersal movements, roaming movements lack the high degree of linearity. The habitual home range of the individual is indicated by a 95 % MCP, which was calculated based on trapping data. For this, we used positions of 42 trapping events collected outside the mating season between 2000 and 2003. (JPEG 279 kb)

ESM 4 Routine movement pathway of a subadult male within its habitual home range. The figure shows a section of the grid system. Illustrated are routine movements (collected during a 40-min observation) for a subadult male during the dry season in the year 2010. Data were collected for the post-dispersal phase of the individual. The habitual home range of the individual is indicated by a 95 % MCP, calculated from 50 independent tracking locations. (JPEG 165 kb)

ESM 5 Movement pathway during three nights of translocation of male E140. **a** Pathway during the first night. The figure shows a section of the grid system. The male was translocated over a distance of 200 m. It gradually increased the explored area using the area around the position where we released it (*black square*) as a base station. **b** Pathway during the second night. The individual headed for the release position (*black square*) less often. Instead, it made a spacious exploration foray away from the release site. Notably, it returned almost on the same pathway to the release area, instead of navigating over a shorter distance. **c** Pathway during the third night. The individual made a foray to the same area as in the night before, using more or less the same pathway. On the way back, the male changed direction and returned to its habitual home range. The change of direction occurred close to the border of the home range of a neighboring male (home ranges indicated as 95 % MCP, calculated based on all available spatial data). (JPEG 465 kb)

ESM 6 Movement pathway during three nights of translocation of male 651E. **a** Pathway during the first night. The figure shows a section of the grid system. The male was relocated over a distance of 300 m. The used area was increased gradually, but the individual remained close to the area where we released it. **b** Pathway during the second night. The individual started to make spacious homing forays away from the release site. Movements strongly resembled dispersal forays. **c** Pathway during the third night. In contrast to dispersal movements, the direction of homing forays was not fixed. (JPEG 528 kb)

## References

[CR1] Alberts SC, Altmann J (1995). Balancing costs and opportunities:dispersal in male baboons. Am Nat.

[CR2] Andreassen HP, Stenseth NC, Ims RA, Bullock JM, Kenward RE, Hails RS (2002). Dispersal behaviour and population dynamics of vertebrates. Dispersal ecology.

[CR3] Baars MA (1979). Patterns of movement of radioactive carabid beetles. Oecologia.

[CR4] Baker RR (1969). The evolution of the migratory habit in butterflies. J Anim Ecol.

[CR5] Barton KA, Phillips BL, Morales JM, Travis JMJ (2009). The evolution of an ‘intelligent’ dispersal strategy: biased, correlated random walks in patchy landscapes. Oikos.

[CR6] Bearder SK, Smutts BB, Cheney DL, Seyfarth RM, Wrangham RW, Struhsaker TT (1987). Lorises, bushbabies, and tarsiers: diverse societies in solitary foragers. Primate societies.

[CR7] Boinski S, Kauffman L, Ehmke E, Schet S, Vreedzaam A (2005). Dispersal patterns among three species of squirrel monkeys (*Saimiri oerstedii*, *S. boliviensis* and *S. sciureus*): I. Divergent costs and benefits. Behaviour.

[CR8] Bonte D, Van Dyck H, Bullock JM, Coulon A, Delgado M, Gibbs M, Lehouck V, Matthysen E, Mustin K, Saastamoinen M, Schtickzelle N, Stevens VM, Vandewoestijne S, Baguette M, Barton K, Benton TG, Chaput-Bardy A, Clobert J, Dytham C, Hovestadt T, Meier CM, Palmer SCF, Turlure C, Travis JMJ (2012). Costs of dispersal. Biol Rev.

[CR9] Bowler DE, Benton TG (2005). Causes and consequences of animal dispersal strategies: relating individual behaviour to spatial dynamics. Biol Rev.

[CR10] Bowman J, Jaeger JAG, Fahrig L (2002). Dispersal distance of mammals is proportional to home range size. Ecology.

[CR11] Byrne RW (1979). Memory for urban geography. Q J Exp Psychol.

[CR12] Clobert J, Ims RA, Rousset F, Hanski I, Gaggiotti O (2004). Causes, mechanisms and consequences of dispersal. Ecology, genetics and evolution of metapopulations.

[CR13] Clobert J, Le Galliard J-F, Cote J, Meylan S, Massot M (2009). Informed dispersal, heterogeneity in animal dispersal syndromes and the dynamics of spatially structured populations. Ecol Lett.

[CR14] Clutton-Brock TH, Lukas D (2012). The evolution of social philopatry and dispersal in female mammals. Mol Ecol.

[CR15] Conradt L, Zollner PA, Roper TJ, Frank K, Thomas CD (2003). Foray search: an effective systematic dispersal strategy in fragmented landscapes. Am Nat.

[CR16] Dammhahn M, Kappeler PM (2008). Comparative feeding ecology of sympatric *Microcebus berthae* and *M. murinus*. Int J Primatol.

[CR17] Dammhahn M, Kappeler PM (2009). Females go where the food is:does the socio-ecological model explain variation in social organisation of solitary foragers?. Behav Ecol Sociobiol.

[CR18] Del Mar DM, Penteriani V, Nams VO, Campioni L (2009). Changes of movement patterns from early dispersal to settlement. Behav Ecol Sociobiol.

[CR19] Dobson FS (1982). Competition for mates and predominant juvenile male dispersal in mammals. Anim Behav.

[CR20] Dufty AM, Belthoff JR, Clobert J, Danchin E, Dhondt AA, Nichols JD (2001). Proximate mechanisms of natal dispersal: the role of body condition and hormones. Dispersal.

[CR21] Eberle M, Kappeler PM (2002). Mouse lemurs in space and time: a test to the socioecological model. Behav Ecol Sociobiol.

[CR22] Eberle M, Kappeler PM (2004). Selected polyandry: female choice and inter-sexual conflict in a small nocturnal solitary primate (*Microcebus murinus*). Behav Ecol Sociobiol.

[CR23] Eberle M, Kappeler PM (2004). Sex in the dark: determinants and consequences of mixed male mating tactics in *Microcebus murinus*, a small solitary nocturnal primate. Behav Ecol Sociobiol.

[CR24] Estes-Zumpf WA, Rachlow JL (2009). Natal dispersal by pygmy rabbits (*Brachylagus idahoensis*). J Mammal.

[CR25] Fietz J (1999). Mating system of *Microcebus murinus*. Am J Primatol.

[CR26] Fredsted T, Pertoldi C, Olesen JM, Eberle M, Kappeler PM (2004). Microgeographic heterogeneity in spatial distribution and mtDNA variability of gray mouse lemurs (*Microcebus murinus*, Primates: Cheirogaleidae). Behav Ecol Sociobiol.

[CR27] Fredsted T, Pertoldi C, Schierup H, Kappeler PM (2005). Microsatellite analyses reveal fine-scale genetic structure in gray mouse lemurs (*Microcebus murinus*). Mol Ecol.

[CR28] Génin F (2003). Female dominance in competition for gum trees in the grey mouse lemur *Microcebus murinus*. Rev Ecol-Terre Vie.

[CR29] Greenwood PJ (1980). Mating systems, philopatry and dispersal in birds and mammals. Anim Behav.

[CR30] Heinz SK, Strand E (2006). Adaptive patch searching strategies in fragmented landscapes. Evol Ecol.

[CR31] Holekamp KE, Sherman PW (1989). Why male ground squirrels disperse: a multilevel analysis explains why only males leave home. Am Sci.

[CR32] Hollander M, Wolfe DA (1999). Nonparametric statistical methods.

[CR33] Hothorn T, Bretz F, Westfall P (2008). Simultaneous inference in general parametric models. Biometrical J.

[CR34] Hothorn T, Hornik K, van de Wiel MA, Zeileis A (2008). Implementing a class of permutation tests: the coin package. J Stat Softw.

[CR35] Isbell LA, Van Vuren D (1996). Differential costs of locational and social dispersal and their consequences for females. Behaviour.

[CR36] Joly M, Zimmermann E (2011). Do solitary foraging nocturnal mammals plan their routes?. Biol Lett.

[CR37] Kappeler PM, Fichtel C, Kappeler PM, Watts DP (2012). A 15-year perspective on the social organization and life history of sifaka in Kirindy Forest. Long-term field studies of primates.

[CR38] Koenig WD, Van Vuren D, Hooge PN (1996). Detectability, philopatry, and the distribution of dispersal distances in vertebrates. Trends Ecol Evol.

[CR39] Kraus C, Eberle M, Kappeler PM (2008). The costs of risky male behaviour: sex differences in seasonal survival in a small sexually monomorphic primate. Proc R Soc Lond B.

[CR40] Larsen KW, Boutin S (1994). Movements, survival, and settlement of red squirrel (*Tarniasciurus hudsonicils*) offspring. Ecology.

[CR41] Lawson Handley LJ, Perrin N (2007). Advances in our understanding of mammalian sex-biased dispersal. Mol Ecol.

[CR42] Lidicker WZ, Stenseth NC, Stenseth NC, Lidicker WZ (1992). To disperse or not disperse: who does it and why?. Animal dispersal: small mammals as a model.

[CR43] Lührs ML, Dammhahn M, Kappeler PM, Fichtel C (2009). Spatial memory in the grey mouse lemur (*Microcebus murinus*). Anim Cogn.

[CR44] Martin A, Bateson P (1993). Measuring behaviour. An introductory guide.

[CR45] Mech LD, Chepko-Sade BD, Halpin ZT (1987). Age, season, distance, direction, and social aspects of wolf dispersal from a Minnesota pack. Mammalian dispersal patterns: the effects of social structure on population genetics.

[CR46] Mittermeier RA, Louis EE, Richardson M, Schwitzer C, Langrand O, Rylands AB, Hawkins F, Rajaobelina S, Ratsimbazafy J, Rasoloarison R, Roos C, Kappeler PM, Mackinnon J (2010). Lemurs of Madagascar.

[CR47] Nunes S, Wolff JO, Sherman PW (2007). Dispersal and philopatry. Rodent societies—an ecological and evolutionary perspective.

[CR48] Pasinelli G, Schiegg K, Walters JR (2004). Genetic and environmental influences on natal dispersal distance in a resident bird species. Am Nat.

[CR49] Pusey AE, Packer C (1987). Dispersal and philopatry. Primate societies.

[CR50] Radespiel U (2000). Sociality in the gray mouse lemur (*Microcebus murinus*) in Northwestern Madagascar. Am J Primatol.

[CR51] Radespiel U, Lutermann H, Schmelting B, Bruford MW, Zimmermann E (2003). Patterns and dynamics of sex-biased dispersal in a nocturnal primate, the grey mouse lemur, *Microcebus murinus*. Anim Behav.

[CR52] Rasoloarison RM, Rasolonandrasana BPN, Ganzhorn JU, Goodman SM (1995). Predation on vetebrates in the Kirindy Forest, Western Madagascar. Ecotropica.

[CR53] Rasoloarison RM, Goodman S, Ganzhorn JU (2000). Taxonomic revision of mouse lemur (*Microcebus*) in the Western portions of Madagascar. Int J Primatol.

[CR54] Rice WR (1990). A consensus combined p-value test and the family-wide significance of component tests. Biometrics.

[CR55] Rodgers AR, Carr AP, Beyer HL, Smith L, Kie JG (2007). HRT: home range tools for ArcGIS.

[CR56] Rogers LL, Chepko-Sade BD, Halpin ZT (1987). Factors influencing dispersal in the black bear. Mammalian dispersal patterns: the effects of social structure on population genetics.

[CR57] Ronce O (2007). How does it feel to be like a rolling stone? Ten questions about dispersal evolution. Annu Rev Ecol Evol Syst.

[CR58] Rood JP, Chepko-Sade BD, Halpin ZT (1987). Dispersal and intertroup transfer in the dwarf mongoose. Mammalian dispersal patterns: the effects of social structure on population genetics.

[CR59] Roper TJ, Ostler JR, Conradt L (2003). The process of dispersal in badgers *Meles meles*. Mammal Rev.

[CR60] Schilling A, Doyle GA, Martin AD (1979). Olfactory communication in prosimians. The study of prosimian behavior.

[CR61] Schmid J (1998). Tree holes used for resting by gray mouse lemurs (*Microcebus murinus*) in Madagascar: insulation capacities and energetic consequences. Int J Primatol.

[CR62] Schmid J, Kappeler PM (1998). Fluctuating sexual dimorphism and differential hibernation by sex in a primate, the gray mouse lemur (*Microcebus murinus*). Behav Ecol Sociobiol.

[CR63] Shields WM, Chepko-Sade BD, Halpin ZT (1987). Dispersal and mating system: investigating their causal connections. Mammalian dispersal patterns—the effects of social structure on population genetics.

[CR64] Smith AT (1974). The distribution and dispersal of pikas:consequences of insular population structure. Ecology.

[CR65] Smith AT, Chepko-Sade BD, Halpin ZT (1987). Population structure of pikas: dispersal versus philopatry. Mammalian dispersal patterns: the effects of social structure on population genetics.

[CR66] Stamps JA, Clobert J, Danchin E, Dhondt AA, Nichols JD (2001). Habitat selection by dispersers: integrating proximate and ultimate approaches. Dispersal.

[CR67] Steen H (1994). Low survival of long distance dispersers of the root vole (*Microtus oeconomus*). Ann Zool Fenn.

[CR68] Storm GL, Andrews RL, Phillips RL, Bishop RA, Siniff DB, Tester JR (1976). Morphology reproduction, dispersal, and mortality of midwestern red fox populations. Wildl Monogr.

[CR69] Sutherland GD, Harestad AS (2000). Scaling of natal dispersal distances in terrestrial birds and mammals. Conserv Ecol.

[CR70] Van Dyck H, Baguette M (2005). Dispersal behaviour in fragmented landscapes: routine or special movements?. Basic Appl Ecol.

[CR71] Waser PM, Austad SN, Keane B (1986). When should animals tolerate inbreeding?. Am Nat.

[CR72] Wiens JA, Clobert J, Danchin E, Dhondt AA, Nichols JD (2001). The landscape context of dispersal. Dispersal.

[CR73] Wiens JA, Zitzmann A (2003). Social structure of the solitary slow loris *Nycticebus coucang* (Lorisidae). J Zool.

[CR74] Yoder JM, Marschall EA, Swanson DA (2004). The cost of dispersal:predation as a function of movement and site familiarity in ruffed grouse. Behav Ecol.

[CR75] Zollner PA, Lima SL (1999). Search strategies for landscape-level interpatch movements. Ecology.

