# Supplementary figures and images for: Walk the line—dispersal movements of gray mouse lemurs (Microcebus murinus)
Source: Behav Ecol Sociobiol. 2012 Jun 26;66(8):1175–85. doi: 10.1007/s00265-012-1371-y (PMC3397133; doi:10.1007/s00265-012-1371-y)

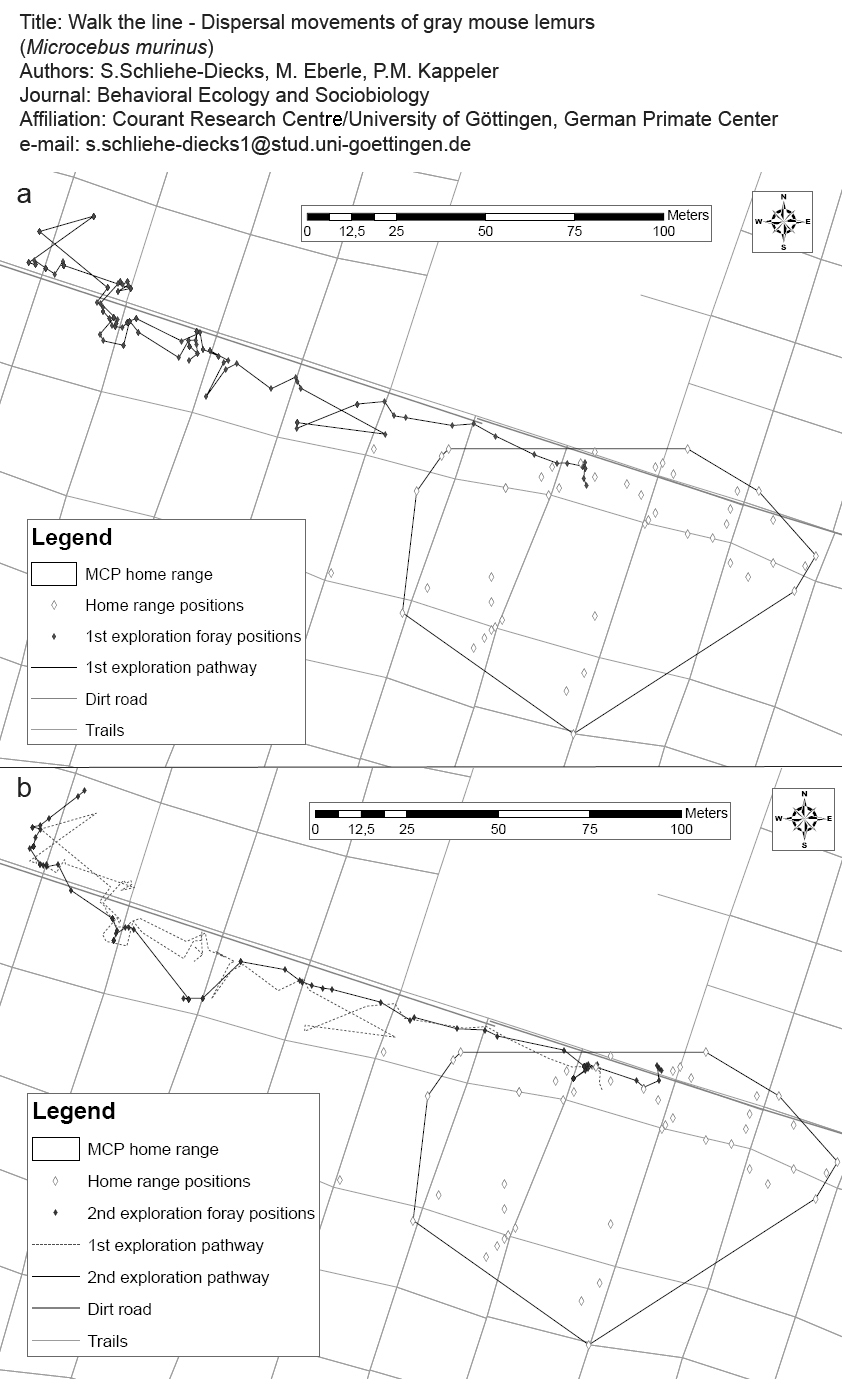

Supplement: Supplementary file 1 — Exploration forays of unsuccessful disperser C404. a First recorded exploration foray. The figure shows a section of the grid system. The natal home range of the individual is indicated by a 95 % MCP, which was calculated based on 50 temporally independent locations (white diamonds) chosen randomly from 196 data points. Gray diamonds show recorded positions during the exploration foray, which we attempted to collect every minute (no. of positions, 127; observation time, 148 min). The solid line connecting them shows the pathway. b Second recorded exploration foray. The second exploration foray occurred 1 week after the first exploration foray. Gray diamonds show again positions during the exploration foray, and the solid line shows the pathway of the second exploration foray (no. of positions, 76; collected within 84 min). The dashed, gray line indicates the route for the first exploration foray. (JPEG 346 kb) [file 265_2012_1371_MOESM1_ESM.jpg]

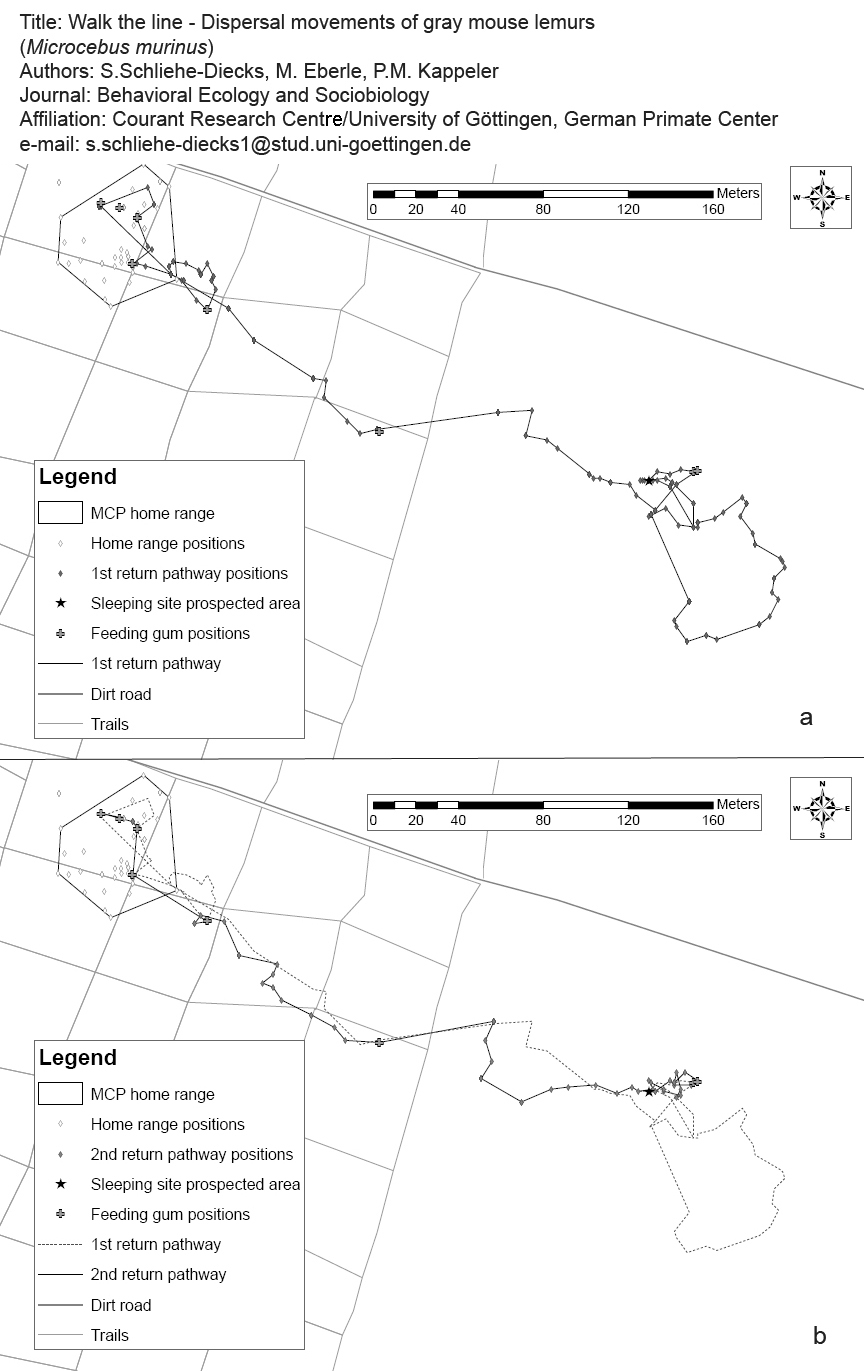

Supplement: Supplementary file 2 — Return pathways of unsuccessful disperser 4B8E. a First recorded return pathway. The figure shows a section of the grid system. The natal home range of the individual is indicated by a 95 % MCP, which was calculated based on 50 temporally independent locations (white diamonds) chosen randomly from 75 data points. The solid line connecting the gray diamonds shows the return pathway (no. of positions, 185). The individual had slept at the prospected site (black star) and was followed from the moment when it started its activity. After initial exploration of the new area, the animal eventually returned to its natal area. Gray crosses represent positions where the individual stopped to feed on gum. The individual was found to have returned back to the prospected site within the same night. b Second recorded return pathway. The second return pathway was recorded the following night. After returning to the prospected area the night before, it used the same sleeping site (black star). Again, we followed the individual from the moment activity started (no. of positions, 81). This was the last incident during which this individual was observed to move outside its natal area. (JPEG 290 kb) [file 265_2012_1371_MOESM2_ESM.jpg]

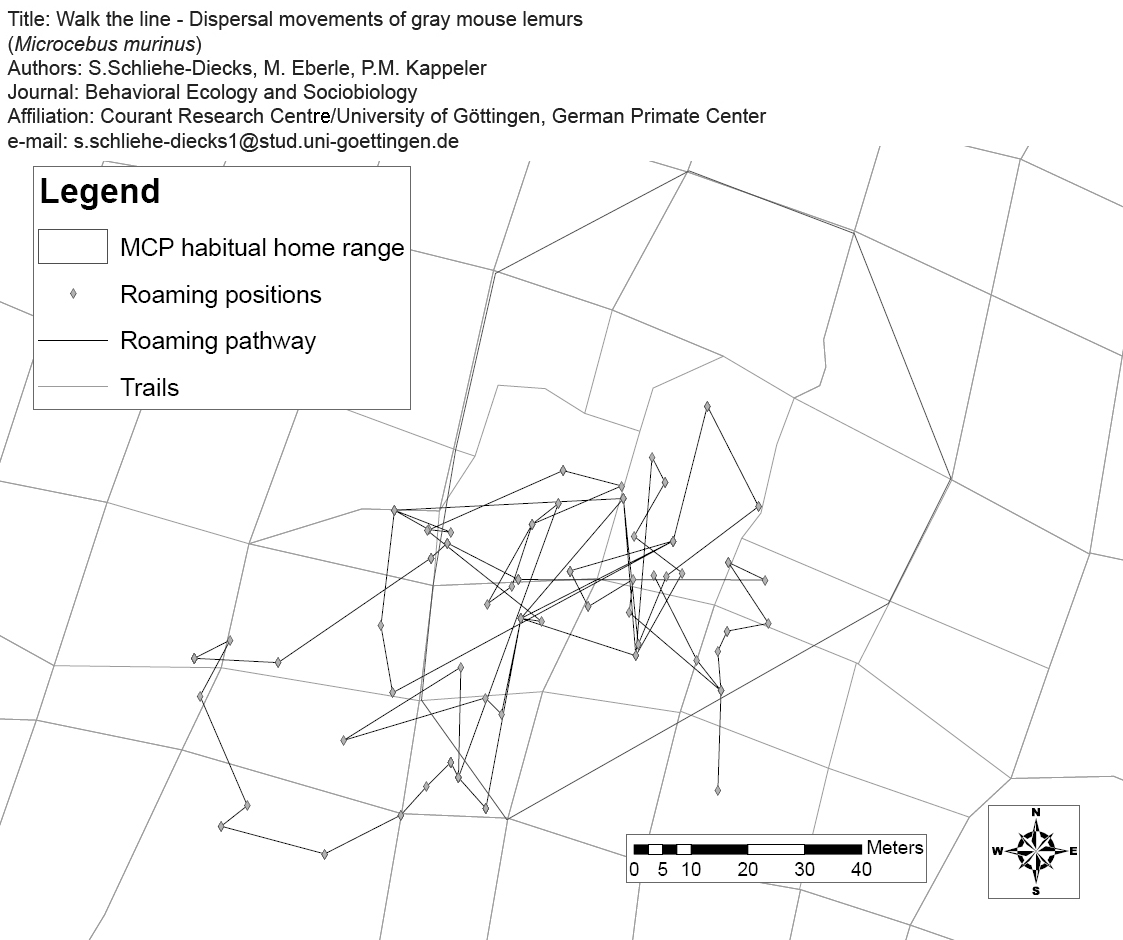

Supplement: Supplementary file 3 — Roaming pathway of a 1-year-old male during mating season. The figure shows a section of the grid system. Illustrated is a pathway of a 1-year-old male during mating season in the year 2000. The male appeared at our study site in April 2000 and was present until October 2003. In contrast to dispersal movements, roaming movements lack the high degree of linearity. The habitual home range of the individual is indicated by a 95 % MCP, which was calculated based on trapping data. For this, we used positions of 42 trapping events collected outside the mating season between 2000 and 2003. (JPEG 279 kb) [file 265_2012_1371_MOESM3_ESM.jpg]

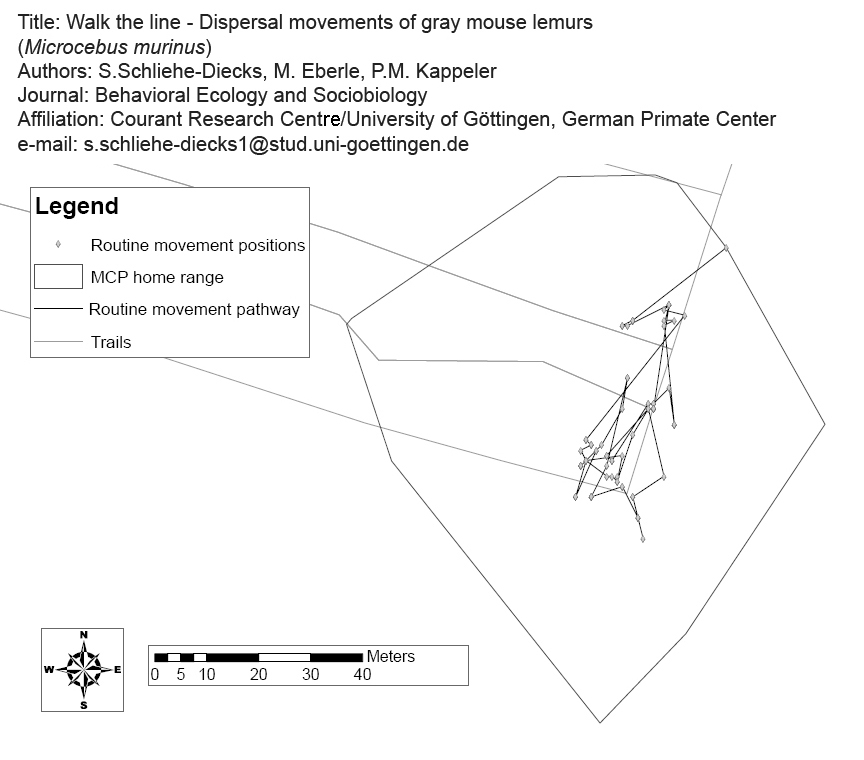

Supplement: Supplementary file 4 — Routine movement pathway of a subadult male within its habitual home range. The figure shows a section of the grid system. Illustrated are routine movements (collected during a 40-min observation) for a subadult male during the dry season in the year 2010. Data were collected for the post-dispersal phase of the individual. The habitual home range of the individual is indicated by a 95 % MCP, calculated from 50 independent tracking locations. (JPEG 165 kb) [file 265_2012_1371_MOESM4_ESM.jpg]

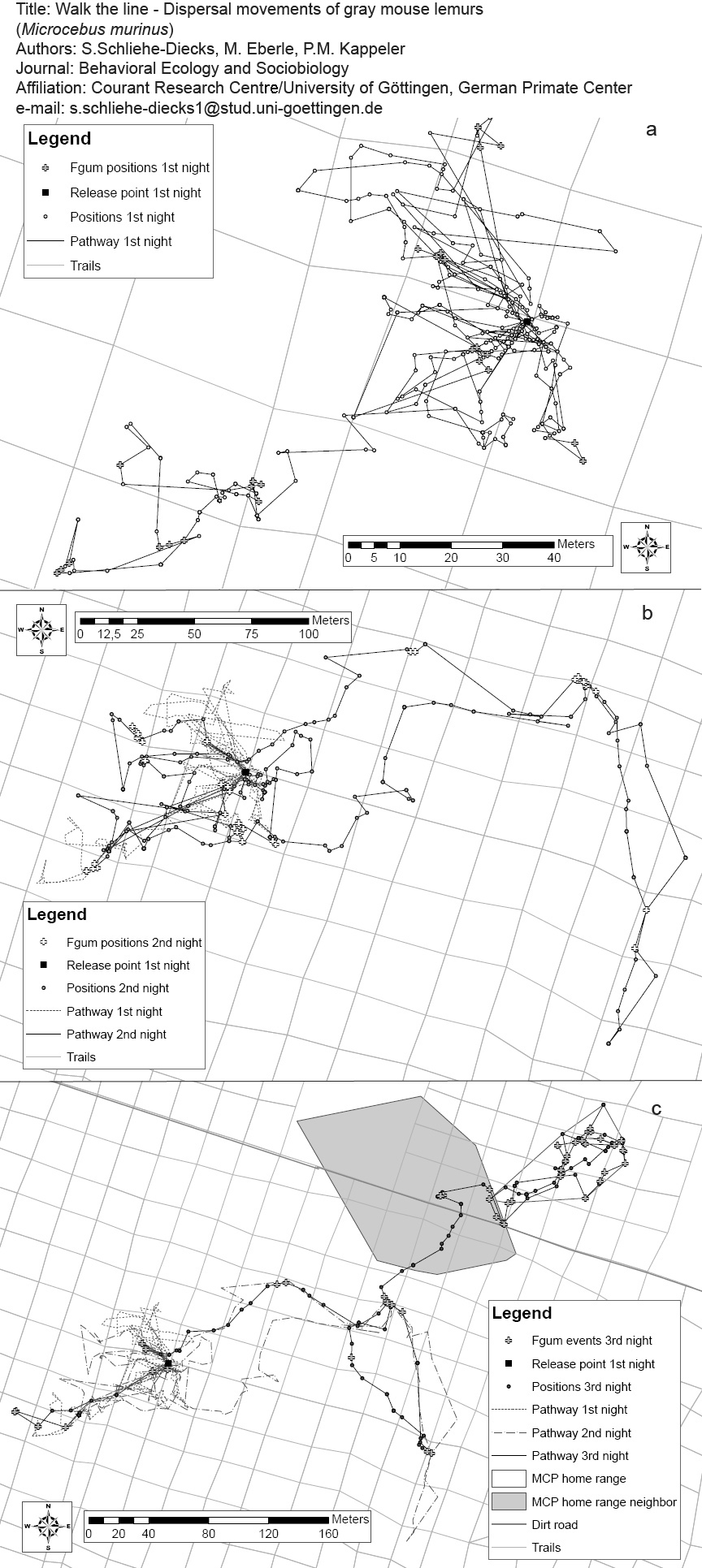

Supplement: Supplementary file 5 — Movement pathway during three nights of translocation of male E140. a Pathway during the first night. The figure shows a section of the grid system. The male was translocated over a distance of 200 m. It gradually increased the explored area using the area around the position where we released it (black square) as a base station. b Pathway during the second night. The individual headed for the release position (black square) less often. Instead, it made a spacious exploration foray away from the release site. Notably, it returned almost on the same pathway to the release area, instead of navigating over a shorter distance. c Pathway during the third night. The individual made a foray to the same area as in the night before, using more or less the same pathway. On the way back, the male changed direction and returned to its habitual home range. The change of direction occurred close to the border of the home range of a neighboring male (home ranges indicated as 95 % MCP, calculated based on all available spatial data). (JPEG 465 kb) [file 265_2012_1371_MOESM5_ESM.jpg]

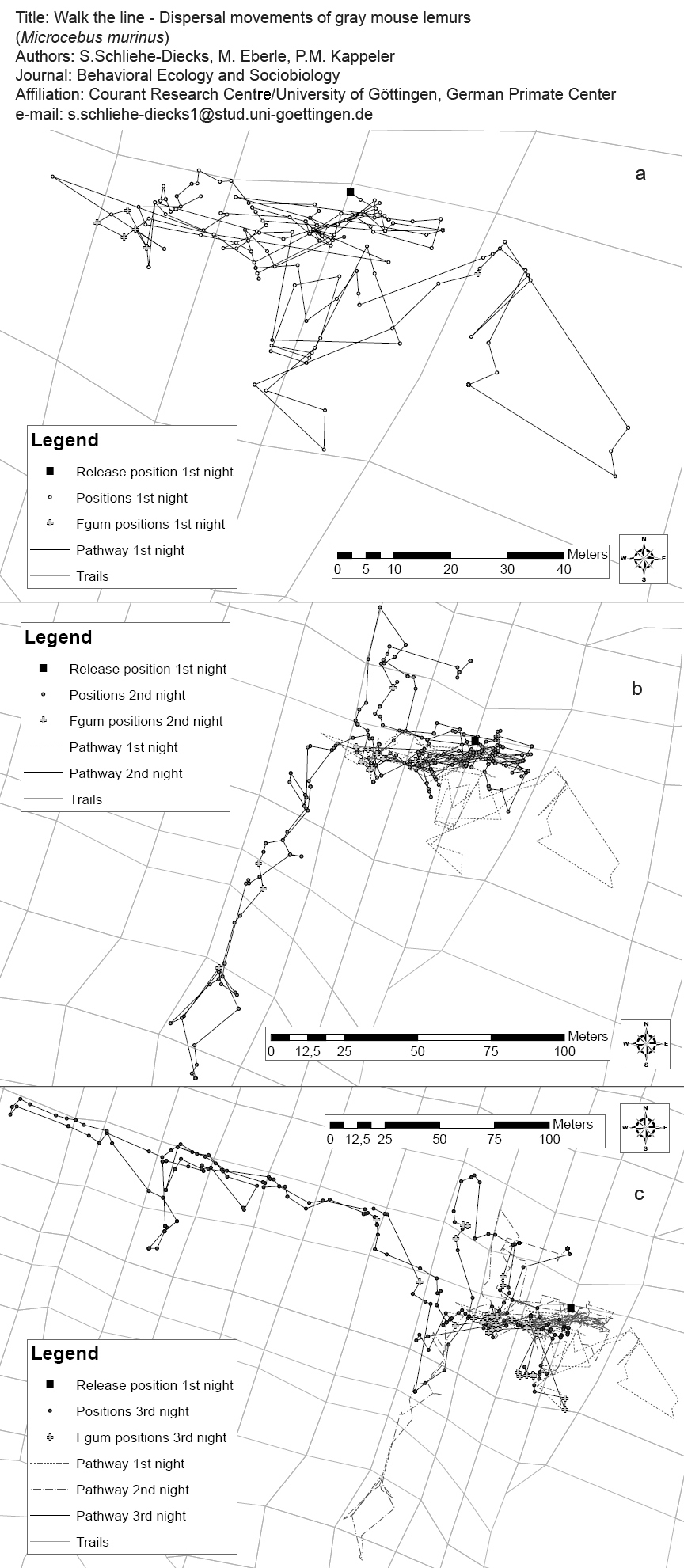

Supplement: Supplementary file 6 — Movement pathway during three nights of translocation of male 651E. a Pathway during the first night. The figure shows a section of the grid system. The male was relocated over a distance of 300 m. The used area was increased gradually, but the individual remained close to the area where we released it. b Pathway during the second night. The individual started to make spacious homing forays away from the release site. Movements strongly resembled dispersal forays. c Pathway during the third night. In contrast to dispersal movements, the direction of homing forays was not fixed. (JPEG 528 kb) [file 265_2012_1371_MOESM6_ESM.jpg]
